# Advanced myocardial characterization and function with cardiac CT

**DOI:** 10.1007/s10554-024-03229-1

**Published:** 2024-09-06

**Authors:** Costanza Lisi, Lukas J. Moser, Victor Mergen, Konstantin Klambauer, Eda Uçar, Matthias Eberhard, Hatem Alkadhi

**Affiliations:** 1 Diagnostic and Interventional Radiology, University Hospital Zurich, University of Zurich, Zurich, Switzerland; 2 Department of Biomedical Sciences, Humanitas University, via Rita Levi Montalcini 4, Pieve Emanuele, 20072 Milan, Italy; 3 Faculty of Medicine, Marmara University, Istanbul, Turkey

**Keywords:** Myocardium, Computed tomography, Photon-counting detector, Late enhancement, Extracellular volume, Strain analysis

## Abstract

Non-invasive imaging with characterization and quantification of the myocardium with computed tomography (CT) became feasible owing to recent technical developments in CT technology. Cardiac CT can serve as an alternative modality when cardiac magnetic resonance imaging and/or echocardiography are contraindicated, not feasible, inconclusive, or non-diagnostic. This review summarizes the current and potential future role of cardiac CT for myocardial characterization including a summary of late enhancement techniques, extracellular volume quantification, and strain analysis. In addition, this review highlights potential fields for research about myocardial characterization with CT to possibly include it in clinical routine in the future.

**Graphical Abstract:**

Cardiac computed tomography as an increasingly important alternative for myocardial tissue characterization and functional assessment.
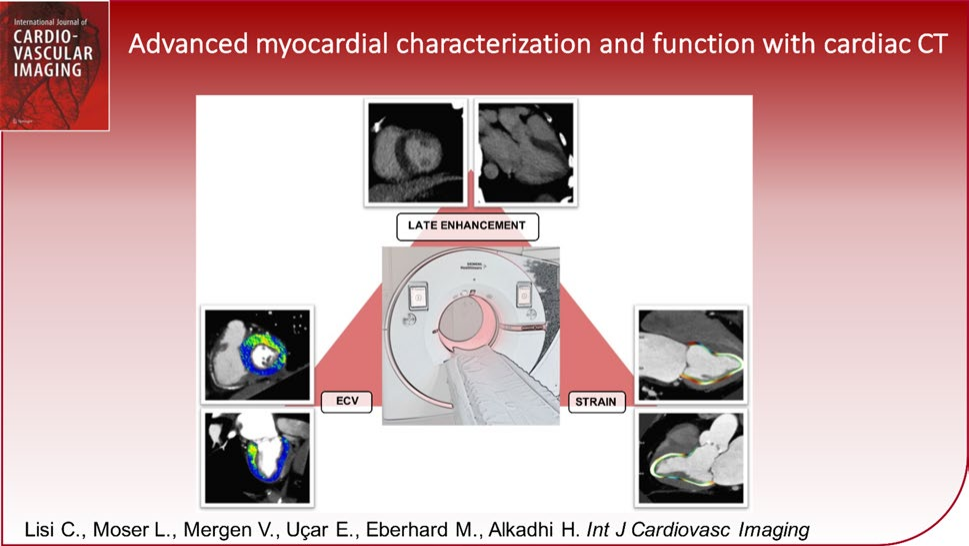

**Supplementary Information:**

The online version contains supplementary material available at 10.1007/s10554-024-03229-1.

## Introduction

Myocardial tissue characterization and functional assessment are crucial for the diagnosis of cardiac disease. Cardiac magnetic resonance imaging (MRI) serves as a cornerstone for the non-invasive assessment of global and regional myocardial tissue composition and function [1]. Presence, extent, and pattern of late gadolinum enhancement (LGE) in cardiac MRI is key to distinguish between ischemic and non-ischemic fibrosis [2] and provides prognostic information [3]. Quantification of extracellular volume (ECV) with cardiac MRI enables the identification of diffuse myocardial fibrosis and aids in prognostication for valvular disease such as aortic stenosis [4]. Myocardial strain (MS) analysis, utilized in both cardiac MRI and echocardiography, facilitates the detection and quantification of subtle wall motion dysfunction, allowing for early diagnosis and robust prognostication in conditions such as ischemic and non-ischemic cardiomyopathy, valvular disease, heart failure, and in cardio-oncology [5–10]. Still, cardiac MRI is not free from limitations: it is a time consuming examination requiring a relatively high level of patient compliance, it may be limited by susceptibility artifact, is contraindicated in patients with certain implants, and may be not feasible in patients with severe claustrophobia.

In recent years, cardiac computed tomography (CT) has emerged as alternative imaging tool for myocardial tissue characterization and functional assessment [11]. Offering availability, robustness, and speed, cardiac CT can be an alternative when cardiac MRI and echocardiography are contraindicated, not feasible, inconclusive or non-diagnostic. Moreover, myocardial tissue characterization with cardiac CT has the potential to become the first line modality for the identification of cardiac amyloidosis in patients with aortic stenosis [12]. Late enhancement imaging is also feasible with cardiac CT to evaluate focal replacement fibrosis, and recent literature underscores the high accuracy of both late enhancement imaging and ECV quantification with CT compared to cardiac MRI [13, 14]. Dual energy (DE) and in particular photon-counting detector (PCD)-CT imaging [15] hold promise for further enhancing the role of CT for myocardial tissue characterization, also eliminating the need for pre-contrast data acquisitions and hereby potentially reducing contrast media volume and radiation exposure to the patients [16]. In addition, recent advancements in CT technology and post-processing will likely further elevate the potential of CT in regard to myocardial deformation analysis [7].

This review aims at a comprehensive summary of current state-of-the-art techniques in myocardial characterization and functional assessment with CT including late enhancement and ECV quantification, and with a special focus on strain analysis, hereby critically assessing the currently existing literature in order to suggest possible future perspectives in this field.

## Late iodine enhancement

Late gadolinium enhancement in cardiac MRI is part of many clinical guidelines and can aid in diagnosis of both acute and chronic myocardial disease [3, 17–19]. Acute myocardial infarction or acute myocarditis show late gadolinium enhancement due to defects in the myocyte membranes allowing for diffusion of contrast media into extracellular space [20]. Chronic processes such as fibrotic myocardial scars, extracellular deposition pathologies, and cardiomyopathies [21–24] lead to fibrotic remodeling of the myocardium with an expansion of the ECV, which can be visualized by late gadolinium enhancement.

Since both gadolinium and iodinated contrast media share similar pharmacokinetic properties [25, 26], the approach of late gadolinium enhancement can be translated also to cardiac CT imaging. Utilizing more advanced CT scanners, Lardo et al. [27] investigated late iodine enhancement scans in a canine model (for acute myocardial infarction 90 min after coronary obstruction) and in a porcine model (chronic state 8 weeks after myocardial infarction). Scans were acquired at multiple timepoints after contrast media injection, and the peak hyperenhancement of the infarcted area was found at 5 min post injection. Authors discovered a strong correlation between histopathologic alterations and the area of late iodine enhancement [27]. Mahnken et al. [28] investigated myocardial viability in acute myocardial infarction in 28 patients with delayed enhancement CT and MRI and found excellent correlation for late enhancement location and size between modalities.

A drawback of late iodine enhancement CT scans, however, remains the relatively lower contrast-to-noise-ratio (CNR) in comparison to late gadolinium MRI [26]. To increase CNR, reduction of tube voltage is an option in patients undergoing single-energy CT [29, 30].However, low tube voltage CT may less effective or not feasible in obese patients due to an increased noise level [31, 32]. Dual-energy (DE) CT may have some advantages for that purpose, allowing for the reconstruction of virtual monoenergetic images (VMI) at low energy levels close to the k-edge of iodine, and the direct quantification of iodine using two-material decomposition techniques. With a rapid kV switching DE approach, Ohta et al. applied these principles in patients with clinically diagnosed heart failure and compared the performance of different VMI levels and iodine maps against MRI [33]. Authors found the highest accuracy for late enhancement in iodine maps on a patient level (96%) and for segment-based evaluation (96%). Oda et al. [34] used a dual layer DECT approach and found excellent agreements between CT and MRI for late enhancement on a segmental level (κ of 0.90 for VMI 50 keV and 0.87 for iodine maps). Chang et al. [35] performed late iodine enhancement scans in cardiomyopathy patients with a dual source CT scanner and found excellent agreement for late enhancement detection (κ 0.97), pattern classification (κ 0.94), differentiation between ischemic and non-ischemic etiology (κ 0.83), and high accuracy (96%) compared to MRI.

The amount of contrast media administered for late iodine enhancement in CT is variable. As a general rule, late iodine enhancement requires a larger volume of administered contrast media to obtain a sufficient contrast-to-noise ratio, which is critical also for ECV calculation. Initially, Bandula et al. used an injection protocol with an iohexol bolus (1 mg/kg at a rate of 3 mL/s) followed by an infusion of 1.88 mL of contrast media/kg/h (with a maximum of 200 mL) and acquired the delayed phase 25 min after the first bolus [30]. Faster protocols were reported by other authors using a fixed bolus of 50–60 mL of iopromide 370 [36], a fixed bolus of 90 mL iohexol 300 [37] or a fixed bolus of 50 mL iopromide 370 for coronary CT angiography followed by an additional bolus of 50 mL of iopromide 370 for late iodine enhancement [38]. Based on the current evidence, fixed volumes of 50–100 mL or weight-dependent volumes of 1.4–1.8 mL/kg of contrast media, especially in obese patients, appear to provide adequate image quality for late iodine enhancement and ECV calculation. A detailed summary of the CT and contrast media protocols for late iodine enhancement can be found elsewhere [39].

Optimal timing of the late enhancement scan remains a topic of active research with no clear consensus in the literature. Many studies report different contrast injection protocols and, depending on the protocol, varying time delays between 3 and 12 min after initial contrast injection. Hamdy et al. [40] performed late iodine enhancement scans 3, 5, and 7 min after contrast injection and reported the highest CNR and best subjective image quality for 5 and 7 min delay. Brodoefel et al. [41] evaluated late iodine enhancement scans at 3, 5, 10, and 15 min and found the best correlation of late enhancement with MRI in the 5 and 10 min late enhancement scans. Jacquier et al. [42] found a higher signal-to-noise-ratio and image quality with a delay of 10 min as opposed to 5 min. The optimal timing may also be influenced by the purpose of late iodine enhancement scans. The visibility of focal fibrosis, e.g. of myocardial scars may be facilitated by longer acquisition delays, as the difference of late iodine enhancement compared to normal myocardium my become more obvious. In contrast, the assessment of diffusely elevated myocardial ECV may be improved by shorter delays as both, attenuation values of the myocardium and the blood pool, are higher due to a higher concentration of iodine compared to longer delays.

Late iodine enhancement scans are usually assessed in a qualitative way. Due to the relatively low CNR, narrow window settings are used to enhance the contrast. Another option to improve the quality has been recently described by Nishii et al., showing how a deep learning-based denoising method supervised with averaged myocardial CT delayed iodine enhancement images improved the CNR [43]. The interpretation of late iodine enhancement images is based on distribution patterns in the myocardium, similar to late gadolinium enhancement. Enhancement patters can be divided into ischemic and non-ischemic. A subendocardial or transmural pattern in vascular distribution is indicative of ischemic cardiomyopathy, whereas mesocardial, subepicardial, and subendocardial patterns not confined to one vessel territory are indicative of non-ischemic cardiomyopathy [35, 44]. Late iodine enhancement CT has also a potential role in patients with acute chest pain. Palmisano et al. [45] showed how a comprehensive CT protocol including a triple rule-out and a late iodine enhancement scan may improve the diagnostic yield in patients with acute chest pain syndrome.

Multicontrast imaging utilizing gadolinium and iodine is an innovative field of current research, with the aim to enable simultaneous evaluation of coronary arteries and myocardium [46]. However, such applications are currently limited to animal studies due to the complexity of translating dual-contrast protocols to human clinical practice. PCD-CT holds promise for the development of novel contrast agents, which may enhance image resolution and diagnostic accuracy by allowing for better differentiation of tissue types and reducing noise and artifacts [47]. Further research is needed to explore the clinical applications of these innovations.

## Extracellular volume

**Fig. 1:**
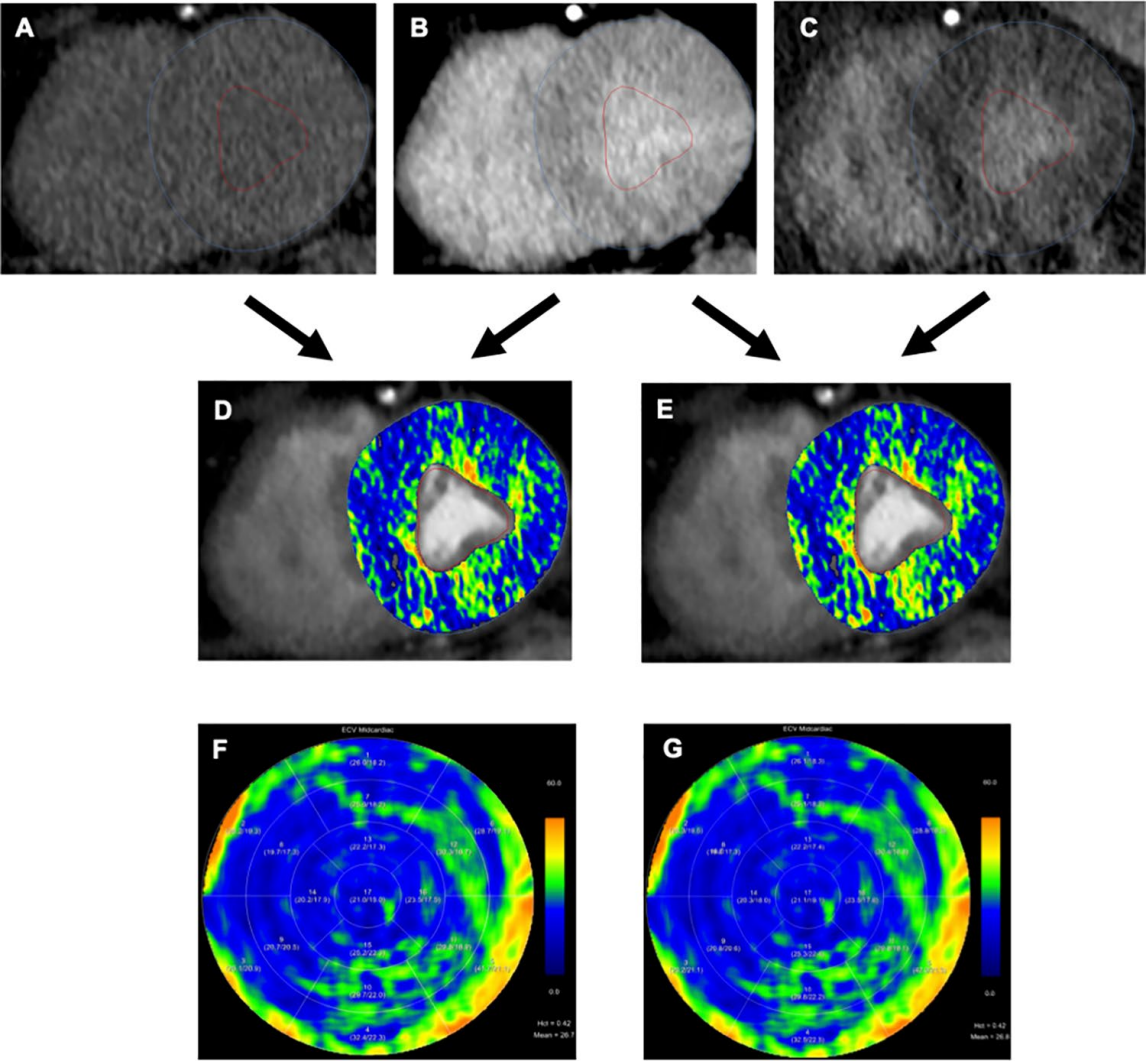
Eighty two-year-old male referred for CT prior to transcatheter aortic valve replacement. CT-derived ECV can be computed using two methods: single-energy attenuation-based and dual-energy spectral-based approaches. For the single-energy method, **A** a true native scan and **B** a late enhancement scan are required, while the native scan is subtracted from the late enhancement scan. For the dual-energy method, **B** a late enhancement scan is used together with **C** an iodine scan. The resulting ECV maps are co-registered and overlaid onto a coronary CT angiography dataset for both the **D** attenuation-based and **E** spectral-based methods. ECV distributions can be visualized using 16-segment polar maps for the attenuation-based (**F**) and spectral-based (**G**) methods

The qualitative evaluation of cardiac late enhancement scans relies on personal expertise of the reader and may be subject to inter-individual variability. Quantification of the myocardial distribution of iodine-based contrast media in the late enhancement phase is also feasible, which is more objective and more reproducible. Myocardial ECV is expressed as a percentage (%) and reflects the proportion of myocardial fibrosis within the myocardium. Two techniques are currently available for calculating myocardial ECV: a single-energy and a dual-energy-based technique [39] (Fig. 1).

The single-energy technique requires two phases for ECV calculations: an ECG-gated non-enhanced scan and an ECG-gated late enhancement scan. The distribution of the contrast media in the myocardium and the blood pool is derived from the attenuation differences, which means a subtraction of the non-enhanced from the late enhancement scan. Attenuation of the blood pool is further adjusted with the blood hematocrit to account for the contrast media concentration of the blood plasma, which is a critical component of the ECV calculation [30, 48–50]. The ECV formula of the single-energy technique is as follows,

$$ECV = \left( {1 - Hct} \right) \cdot \frac{{\Delta HU_{myocardium} }}{{\Delta HU_{bloodpool} }}$$.

When utilizing the dual-energy technique, ECV calculation necessitates only one cardiac scan phase: an ECG-gated cardiac late enhancement phase. Spectral information contained in the dual-energy late enhancement scan enables the reconstruction of iodine maps and the direct deduction of the concentration of the contrast media within the myocardium ($[Iodine_{myocardium} ]$) and the blood pool ($[Iodine_{bloodpool} ]$) [34, 51–53] (see Fig. 1). The ECV formula of the dual-energy technique is as follows,

$$ECV = \left( {1 - Hct} \right) \cdot \frac{{[Iodine_{myocardium} ]}}{{[Iodine_{bloodpool} ]}}$$.

The dual-energy based approach appears to be more robust and increases the number of studies being feasible for ECV calculation [52]. In contrast to the single-energy technique, it is not susceptible to misregistration of the non-enhanced and late enhancement scans caused by differences in cardiac phases, motion artifacts due to irregular heart rates, or different breath-hold levels. Several protocols for late enhancement CT to quantify the ECV have been tested. Typically, single-energy CT-based ECV is calculated acquiring a non-enhanced scan, a CT angiography scan, and a delayed phase scan. A delayed scan 10 min post contrast administration was proposed by an early study [29], while others tested different delays (3, 5, and 7 min) [40]. No significant differences in ECV values were detected at these time points, but scans at 5 and 7 min provided better scar identification [40]. For dual-energy protocols, late enhancement scans were acquired from 5 to 12 min post contrast media injection [38, 54, 55]. Recent evidence suggests that 3-min delay improves normal myocardium ECV quantification, while a 5-min delay enhances scar visualization [39].Several studies have assessed the accuracy of myocardial ECV determined by CT with the invasive reference standard, endomyocardial biopsy, and the imaging reference standard, tissue characterization with cardiac magnetic resonance imaging [30, 39, 49, 56]. Bandula et al. [30] investigated the extracellular volume determined by CT in 23 patients with severe aortic stenosis, finding a significant correlation with histological extracellular fibrosis (*r* = 0.71, *p* = 0.0007) and ECV determined by MRI (*r* = 0.73, *p* < 0.0002). In a retrospective study conducted by Treibel et al. [49] involving 26 patients with systemic amyloidosis and 27 patients with severe aortic stenosis, ECV determined on cardiac late enhancement CT scans acquired 5 min after contrast media injection demonstrated a higher correlation than using scans acquired after 15-min delay, with cardiac MRI serving as the reference standard. When considering patients with nonischemic cardiomyopathy and heart failure, ECV determined by CT also demonstrated good agreement with ECV determined by cardiac MRI [54, 57, 58]. For a detailed overview see [39].

**Fig. 2:**
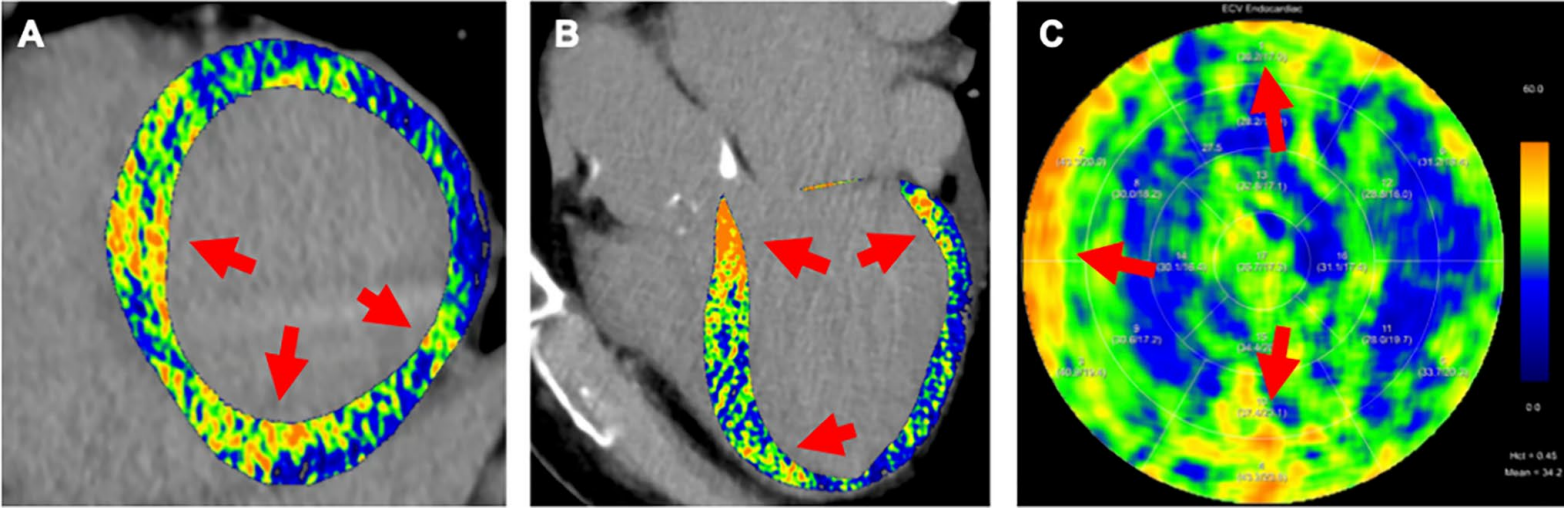
Seventy-five-year-old female patient with confirmed cardiac transthyretin amyloidosis. CT-derived ECV maps are overlaid on late enhancement CT images in **A** short-axis and **B** three-chamber views and are represented in the **C** polar map. Red arrows highlight areas with pronounced myocardial injury. The mean myocardial ECV was elevated at 31.74% (normal: 20–26%)

**Fig. 3:**
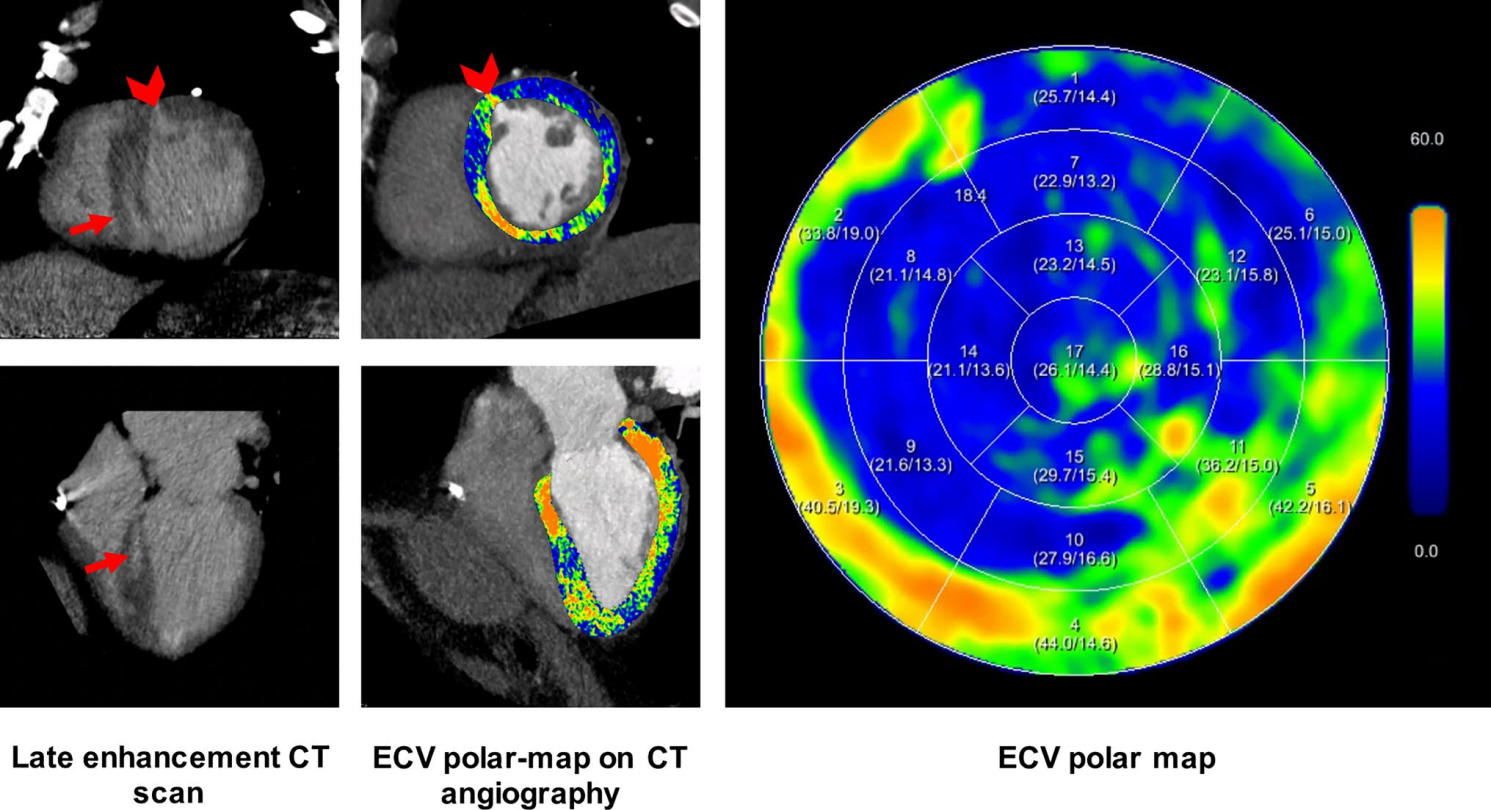
Eighty two-year-old female patient with severe aortic stenosis and known chronic ischemic cardiomyopathy. Late iodine enhancement images and iodine maps document increased myocardial late enhancement in the basal infero-septal left ventricular wall (red arrow in late enhancement CT). Elevated ECV values (patient hematocrit 43%) are shown in the corresponding territory. In addition, a tiny area of myocardial late enhancement is seen at the basal antero-septal junction (arrowhead in short axis late enhancement CT and ECV polar map), which may be related to aortic stenosis. Elevated ECV values are also shown in the basal infero-lateral segment, as the result of a known previous myocardial infarction

**Fig. 4:**
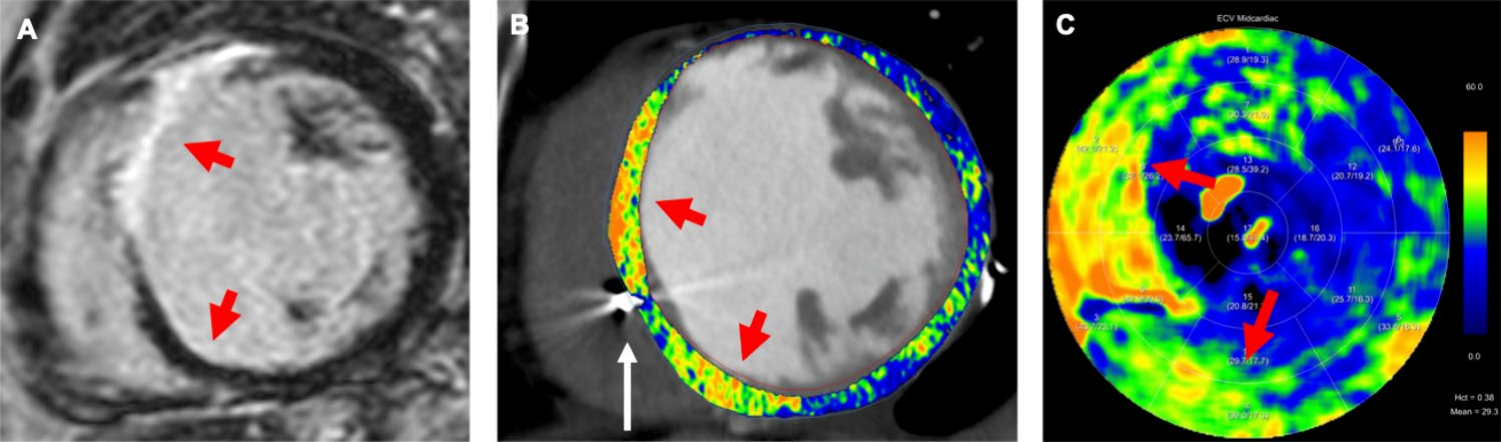
Seventy four-year-old female patient with chronic myocardial infarction after occlusion of the left anterior descending artery. Red arrows indicate antero-septal transmural and infero-septal subendocardial scar in late gadolinium enhancement MRI in the **A** short-axis view. Extracellular volume overlaid onto coronary CT angiography in the **B** short-axis and in a **C** polar map. White arrow shows a pacemaker lead in the right ventricle **B** inserted after MRI due to arrhythmia

**Fig. 5:**
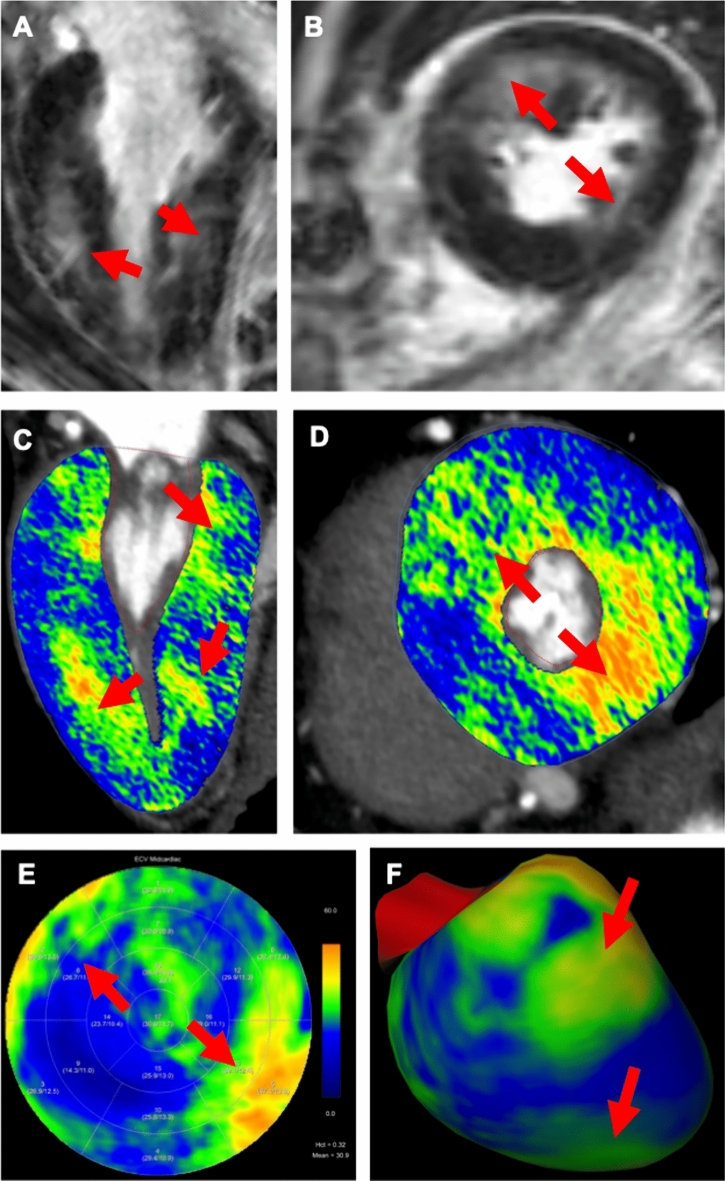
Sixty nine-year-old male patient with hypertrophic cardiomyopathy. Red arrows indicate typical basal accentuated left ventricular hypertrophy with late gadolinium enhancement at right ventricular insertion points in cardiac MRI in **A** two-chamber and **B** short axis views. Corresponding extracellular volume elevations are shown also on **C** two-chamber and **D** short-axis views in CT, additionally represented in the 16-segment **E** polar map and on a **F** three-dimensional heart model

Importantly, non-invasive quantification of myocardial ECV by CT has been identified as an independent prognostic marker for various pathological conditions [4, 37, 55, 59–63]. The extent of myocardial fibrosis, as reflected by ECV, is associated with the outcomes of patients undergoing transcatheter or surgical aortic valve replacement for severe aortic stenosis [4, 37, 55, 59–61]. In recent studies by Koike et al. [61] and Vignale et al. [64], authors observed that a septal ECV derived using the single-energy technique equal to or higher than 28.5 or 31.3%, respectively, was associated with a higher risk of all-cause mortality and heart failure hospitalization. Moreover, elevated ECV values have been described to correlate with adverse cardiac remodeling and all-cause mortality in patients with confirmed systemic amyloidosis [62] (Fig. 2), and to serve as a predictor for major cardiovascular events in patients with dilated cardiomyopathy [65]. Representative further examples of late iodine enhancement and ECV imaging with CT are shown in Figs. 3, 4 and 5.

Conventional dual-source CT scanners with energy-integrating detectors (EID), when operated in the dual-energy mode, do not exploit the full (maximum) temporal resolution of the scanner compared to the single-energy mode. This limitation in temporal resolution to that of a single-source CT scanner can be relevant in patients with higher or irregular heart rates, as it may lead to motion artifacts and reduced image quality hereby affecting the accuracy of ECV measurements [6]. The advent of PCD-CT into routine clinical practice heralds a new era for spectral and quantitative cardiac CT imaging. Since multi-energy separation is achieved at the detector, dual-source cardiac PCD-CT can combine inherent spectral resolution with high temporal resolution [52, 67]. Aquino et al. [67] demonstrated a high correlation between ECV quantification, employing the dual-energy technique, and tissue characterization with cardiac MRI [67].

Recent blood hematocrit values are crucial for calculating myocardial ECV, as they correct iodine concentration within the blood to iodine concentration in the blood plasma. However, these values are not always available due to sampling errors or outpatient settings during which blood tests are not routinely obtained. Decker et al. [68, 69] indicated that blood pool attenuation derived from virtual non-contrast images with PCD-CT exhibited a high correlation with blood hematocrit. Building on this concept, Mergen et al. [70] recently showed with PCD-CT that the hematocrit can be calculated from late iodine enhancement CT images, hereby obviating the need for taking blood samples in each patient. Nevertheless, synthetic hematocrit calculations might be not perfectly accurate, since they are subject to variations introduced by image noise and other patient-specific factors. Moreover, synthetic hematocrit might not be universally applicable across different CT scanners and protocols, limiting its widespread adoption [70, 71]. Further validation against traditional hematocrit measurements are essential. The increasing availability of spectral CT machines including PCD-CT may further enhance the accuracy and clinical value of this technique in the near future. Spectral data may lead to CT-based ECV calculation with a lower contrast media volume and lower radiation dose making this technique the standard for prognostication in selected patient populations, such as those with severe aortic stenosis being candidated to trans catheter valve replacement [4].

## Strain analysis

### General principle

MS quantifies the percentage of deformation in a myocardial segment, calculated as the difference between its maximum length during end-systole (L_max_) and its initial length during end-diastole (L0), expressed by the formula:

$$\left( {{\mathrm{L}}_{{{\mathrm{max}}}} - {\mathrm{L}}0} \right)/{\mathrm{L}}0$$

Imaging-based MS analysis relies on Lagrangian strain definition, where myocardial shift is computed at a given point, with deforming myocardium serving as the displacement reference [72]. MS analysis decomposes myocardial deformation into three primary components: radial strain, circumferential strain, and longitudinal strain [73]. Radial strain represents the relative myocardial thickening towards the LV cavity center; longitudinal strain indicates fiber shortening over the cardiac long axis, and circumferential strain represents myocardial fiber shortening on a cardiac short-axis [74]. Additionally, the rate of deformation along these axes can be calculated, such as circumferential-longitudinal, circumferential-radial, and longitudinal-radial strain rates. MS analysis can be performed using various tracking techniques. The speckle-tracking technique uses ultrasound speckles as indirect index of myocardial deformation [75]. The feature-tracking (FT) technique, mostly employed in cardiac MRI and CT, tracks endocardial and epicardial border deformation to estimate MS [76]. More specifically, FT CT algorithms track endo- and epicardial specific points throughout the cardiac cycle by identifying and following distinct myocardial tissue patterns or features in successive CT images. The displacement of these tracked features is analyzed to compute CT-based MS.

#### Data from echocardiography and MRI

Substantial literature supports the utility of MS analysis with cardiac MRI and echocardiography for diagnosis and prognostication across a diverse range of cardiac disease. In the realm of ischemic cardiomyopathy, speckle tracking echocardiography analysis serves multiple purposes, aiding in the early detection of post-ischemic dysfunction [77], predicting significant coronary artery disease (CAD) in stable angina [78], and estimating infarct size in non-ST elevation myocardial infarction [79]. Non-ischemic cardiomyopathy also benefits from MS analysis application, where global longitudinal strain (GLS) alterations precede changes in ejection fraction (EF) in subclinical hypertrophic cardiomyopathy (HCM) [19]. Myocardial deformation analysis is also part of the diagnostic criteria for arrhythmogenic cardiomyopathy (ACM), in both the 2010 Task Force Criteria [80] and the 2020 Padua criteria [17]. Furthermore, MS analysis is integral to cardio-oncology, as recommended by the recent 2022 European Society of Cardiology (ESC) cardio-oncology guidelines, advising adding GLS analysis to MRI workup for patients suspected of cardiotoxicity [81]. Beyond these applications, myocardial deformation imaging demonstrates added clinical value in valvular heart diseases [82–85] and holds promise in the diagnosis of acute myocarditis [86, 87].

#### Data from myocardial strain analysis with CT

**Fig. 6:**
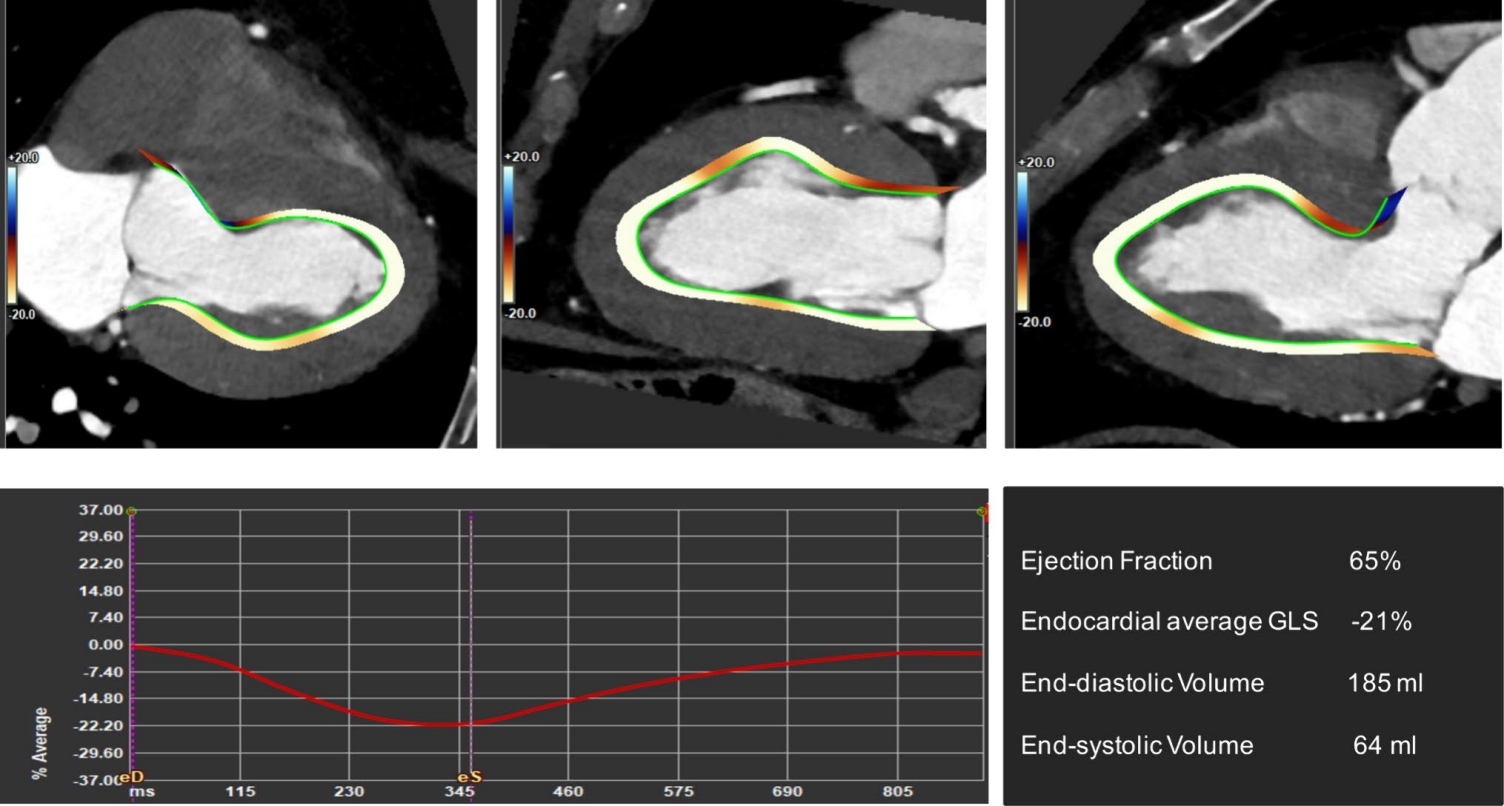
4D CT derived Global Longitudinal Strain (GLS). 67-year-old-male patient with mild aortic stenosis. Positive and negative endocardial end-systolic GLS values are marked in blu and red in the long-axis view (top line). The average GLS-time curve is presented (bottom left) with the prinicpal functional and volumetric parameters derived from the analysis (bottom right) showing normal GLS (-21%) and a normal ejection fraction (65%)

**Fig. 7:**
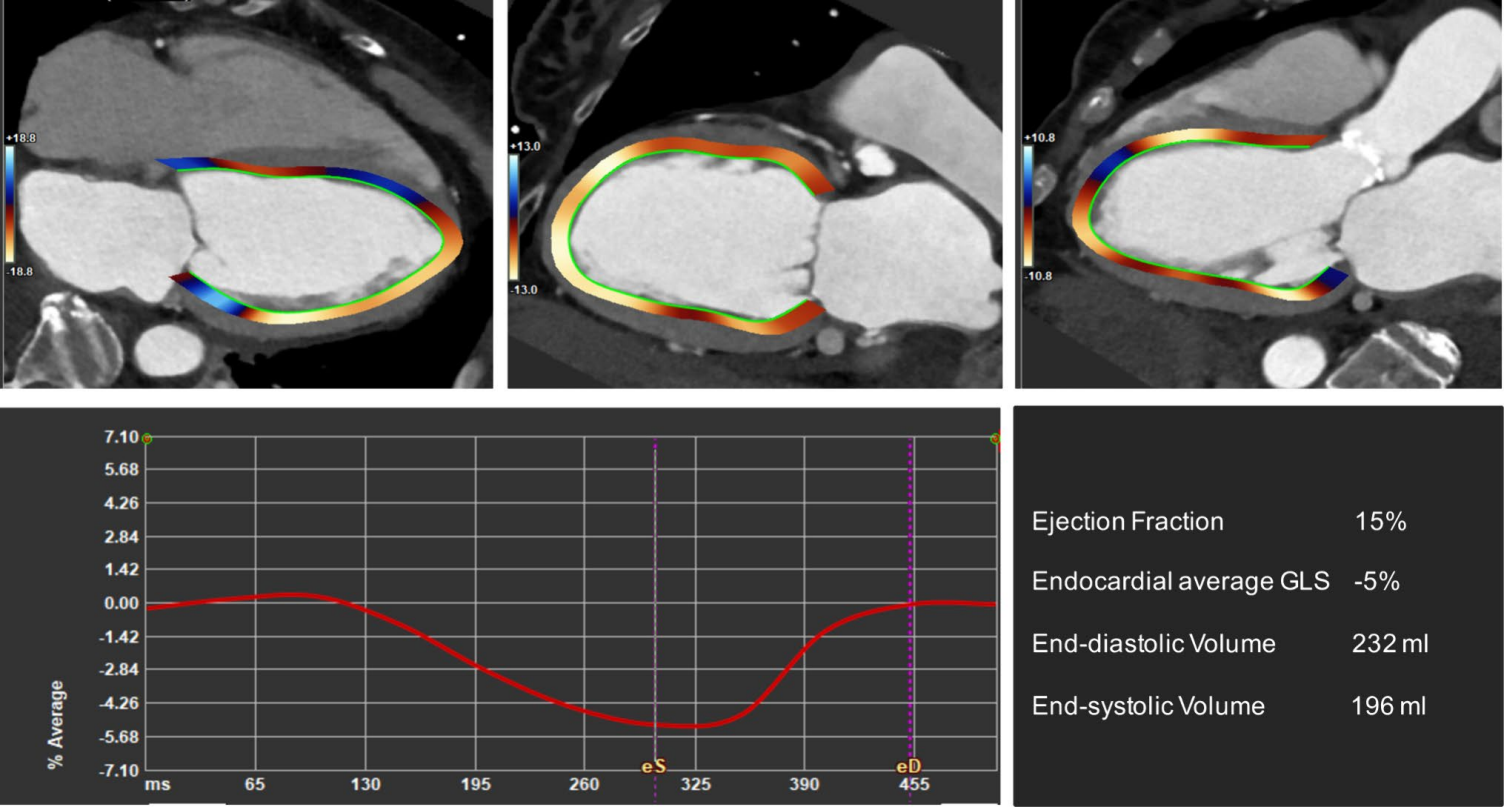
4D CT derived Global Longitudinal Strain (GLS). 74 year-old-female patient with severe aortic stenosis (see aortic calcifications in 3-chamber view, top line). Positive and negative endocardial end-systolic GLS values are marked in blu and red in the long-axis view (top line). The average GLS-time curve is presented (bottom left) with the prinicpal functional and volumetric parameters derived from the analysis (bottom right) showing abnormal average GLS (− 5%), strongly reduced ejection fraction (15%), and a severely dilated left ventricle

CT-derived MS analysis may serve as a viable alternative to both speckle tracking echocardiography and cardiac MRI analysis in cases of contraindications, poor acoustic window, and challenges in evaluating right ventricular (RV) and left atrial (LA) strain. Several studies have examined CT-derived MS analysis against echocardiography- and MRI-based myocardial deformation analysis. Good correlation in strain values was found between echocardiography and CT in patients screened for cardiovascular disease [88], patients with aortic stenosis [89], and in patients with heart failure [90]. Strong correlation was found comparing CT- and MRI-derived MS analysis [7, 12, 88–95]. Tee et al. [92] showed that CT regional strain analysis effectively detects focal dysfunction in a swine experimental model of cardiomyopathy with acceptable comparability to MRI. In vivo studies confirmed the agreement between myocardial deformation measures derived from CT and those from echocardiography and MRI [91]. Li et al. [95], found a significant correlation of global MS with coronary artery calcium score and semi-quantitative coronary stenosis assessment with CT, suggesting that GLS may be a suitable alternative to calcium scoring for assessing CAD severity. In patients planned to undergo transcatheter aortic valve replacement (TAVR) employing a 4D CT protocol [96], excellent reproducibility of CT-based strain measures were demonstrated [7]. Additionally, CT strain measures exhibited high accuracy in identifying transthyretin amyloid cardiomyopathy compared to the reference standard technetium 99 m 3,3-diphosphono-1,2-propanodicarboxylic-acid scintigraphy [12] and to predict short-term outcomes after TAVR [97]. Representative examples of CT strain analyses are provided in Figs. 6 and 7 and in the Online resources 1–6.

#### CT protocols and post-processing software

The literature on CT-based MS analysis is relatively heterogeneous in regard to applied CT techniques, contrast media and CT protocols, and post-processing algorithms. Table 1 provides a summary of the existing literature on CT-based MS analysis, detailing scan and contrast media protocol, reconstruction techniques, and software utilized. Scanner specific temporal resolution ranged between 60 ms [92] to 175 ms [93]. Tube voltage ranges from 80 to 120 kV, often tailored to patient size [7, 12, 88–95], with ECG-based dose modulation commonly applied. High concentration contrast media is typically administered (320–400 mgI/ml), with the bolus tracking technique employed to start data acquisition. Most studies utilized a single bolus administration, although exceptions exist, such as Manohar et al. [93] who employed a triphasic bolus technique. Reconstruction increments of 5–10% throughout the entire cardiac cycle are utilized. Various reconstruction slice thicknesses (ranging from 0.5 mm to 0.75 mm) and increments (ranging from 0.4 to 1 mm) are applied across studies. Effective radiation doses are variable and range from 2.8 mSv [93] to 15.8 mSv [90].

**Table 1:** Summary of CT studies evaluating strain

	Scanner type (vendor)	Temporal resolution (ms)	Tube voltage (kV)	ECG-pulsing	Scan mode/ ECG-gating	Contrast media (mgI/ml)	Contrast media dose & flow rate	RR acquisition interval	Time interval	Slice thickness/ increment	Reconstruction algorithm	Strain analysis technique (software)	Radiation dose a DLP Effective dose	Agreement with other modalities (*p* < 0.05)
Tavakoli 2014 [91]	64-slice single-source (Siemens)	160	100/120	No	Retrospective	Iomeprol 400	70-80 ml 5 ml/s	0–95%	5%	Not specified	Not specified	Landmark Tracking with an in-house tool	1550 mGy cm 21.7mSv^a^	Echo GRS r = 0.67 GCS r = 0.61 MRI GRS r = 0.76 GCS
Buss 2014 [90]	2 × 64-slice dual-source (Siemens)	60	120	Yes	Retrospective	Iopromide 370	80 ml 5 ml/s	0–95%	5%	0.75 mm/not specified	Medium smooth kernel (B26f)	Feature tracking software (2D CPA MR prototype, TomTec Imaging Systems)	15.8 ± 8 mSv	Echo GRS r = 0.93 GCS r = 0.87 GLS r = 0.84
Tee 2015 [92]	320-slice single-source (Toshiba)	137	120	not specified	Retrospective	Iodixanol 320	60 ml 5 ml/s	0–100%	5%	Not specified	Iterative reconstruction: AIDR3D standard	Multimodality tissue-tracking software (Toshiba)	Not specified	MRI GRS r = 0.55
Manohar 2022 [93]	320-slice single-source (Canon)	175	100/120	Yes	Retrospective	Iopamidol 370	55 ml 5 ml/s	Not specified	Not specified	0.5 mm/not specified	Cardiac FC03 kernel	Point-tracking with an in-house tool	198.4 ± 134.6 mGy cm 2.8 mSv	MRI GCS r = 0.40 GLS = 0.64
Ammon 2019 [89]	2 × 192 slice dual-source (Siemens)	66	100	Yes	Retrospective	Iopromide 370	50 ml 5 ml/s	0–100%	10%	0.75 mm/ 0.5 mm	Medium soft kernel (Bv40) ADMIRE level 2	Feature-tracking (Ziostation2, Ziosoft Inc., Tokyo, Japan)	Not specified	Echo GLS r = 0.71
Wang 2021 [94]	2 × 128-slice dual-source (Siemens)	75	80/100	-	Prospective sequential	Iohexol 350	60-90 ml 4.5–5 ml/s	0–100%	5%	0.6 mm/0.4 mm	Iterative reconstruction: factor 3	Feature-tracking (CVI software version 5.11, CVI Circle Cardiovascular Imaging)	387.86 ± 89.3 mGy cm 5.43 mSv	MRI GRS r = 0.89 GCS r = 0.86 GLS r = 0.79
Kinoshita 2021 [88]	2 × 192 slice dual-source (Siemens)	66	100	Yes	Retrospective	Iopamiron 370	50 ml 5 ml/s	0–95%	5%	0.75 mm/0.4 mm	Medium soft kernel (Bv40) ADMIRE level 2	Feature-tracking (Ziostation2, Ziosoft Inc., Tokyo, JaSvn)	6.2 ± 2.4 mSv	Echo GRS r = 0.43 GCS r = 0.65 GLS r = 0.74
Bernhard 2022 [7]	2 × 128-slice dual-source (Siemens)	75	100/120	Yes	Retrospective	Not specified	40–120 ml 4–5 ml/s	0–100%	5%	Not specified/1 mm	Medium smooth kernel (I30f) SAFIRE level 3	Feature-tracking (Medis Suite QStrain)	957 mGy cm 13.4mSv^a^	Echo GRS r = 0.39 GCS r = 0.40 GLS r = 0.81
Bernhard 2023 [12]	Somatom definition flash (Siemens)	75	100/120	Yes	Retrospective	Iopromide 370 Single bolus	1 ml/kg 4–5 ml/s	0–100%	5%	Not specified/1 mm	Medium smooth kernel I30f SAFIRE level 3	Feature-tracking (Medis Suite QStrain)	973.0 ± 415.6 mGy cm 13.6mSv^a^	Not specified
Li 2023 [95]	Somatom force (Siemens)	66	120	Yes	Retrospective	Iopromide 400 Single bolus	30–60 ml 2–4 ml/s	0–100%	5%	0.75 mm /0.5 mm	Medium soft kernel (Bv40)	Feature-tracking (Medis Suite QStrain)	4.9 ± 1.4 mSv	Not specified

^a^If not provided in the paper, effective radiation dose (in mSv) was calculated using a conversion coefficient of 0.014 mSv/(mGy cm^2^)

*DLP* Dose length product

MS analysis is performed using different techniques and software solutions. While Tavakoli [91] and Manohar [93] developed in-house tools for landmark and point-tracking of the myocardium, others utilized commercially available software [7, 12, 88–95].

#### Open issues that need be addressed in future studies

While the recently growing body of evidence supports the application of CT for MS analysis, several issues need be addressed in future studies. The impact of temporal resolution on MS values accuracy remains poorly investigated. Studies with echocardiography and MRI demonstrated that temporal resolution had relevant impact on MS analyses [98]. Similarly, RR reconstruction increments may also influence the results from CT-based MS analysis, as suggested by a recent study comparing 5 and 10% increments [99]. Thus, further validation studies are needed to define a standardized CT protocol and homogenous reconstruction settings before a widespread application can be advocated.

Another important issue of MS analyses with CT is the applied radiation dose, given that the entire cardiac cycle needs to be covered, resulting in relatively higher radiation doses compared to sequential scans with coverage of only parts of the cardiac cycle or high-pitch spiral CT acquisitions. Doses varied considerably across studies (see Table 1), and methods to keep dose at as low as reasonably achievable need to be evaluated and standardized. In addition, inter-platform robustness needs to be proven too to improve reproducibility. Challenges in LV endocardial border drawings at the long axis apical level in hypertrophic patients, as well as variable definition of the LV basal, mid and apical level on short-axis images may be responsible for inter-observer and inter-platform variations and need to be addressed for enabling robust clinical application. Here, CT has the advantage of providing a true three-dimensional dataset with isotropic resolution for evaluating myocardial contractility. Additionally, indications for myocardial deformation analysis with CT should be defined in order to appropriately define its role in routine clinical practice.

## Outlook and conclusion

Offering availability, robustness, speed and—in recent years—also improved soft tissue contrast and dual-energy capabilities particularly through the introduction of PCD technology, cardiac CT has emerged as alternative imaging technique for myocardial tissue characterization and assessment of function. Thus, CT can be considered a valuable alternative when cardiac MRI and/or echocardiography are contraindicated, not feasible or non-diagnostic. Particularly recent approaches for ECV quantification and strain analysis hold promise as a future mainstay in cardiac CT imaging, provided standardization efforts will take place to homogenize scan and contrast media protocols as well as post-processing algorithms and techniques. Further emerging techniques for tissue characterization such as radiomics, texture analysis, and machine learning may further improve myocardial tissue characterization. For instance, the impact of iterative reconstructions on texture analysis and its capability of scar tissue detection has been recently demonstrated [100], as well as its application to identify myocardial infarction in low-dose non-contrast cardiac CT images used for calcium scoring [101]. Machine learning-supported data analysis can also detect myocardial edema and fatty infiltration, providing insights into inflammation and cardiomyopathy [102]. Overall, these emerging techniques have the potential to significantly contribute to the field of CT-based myocardial characterization, enhancing diagnostic accuracy, treatment planning, and ultimately improving patient outcomes.

## Supplementary Information

Below is the link to the electronic supplementary material.

4D CT derived 2-chamber Global Longitudinal Strain (10 cardiac phases). 67-year-old-male patient (see Fig. 2) with mild aortic stenosis with a GLS of − 21% and an ejection fraction of 65%.
Supplementary file1 (MP4 4283 KB)

4D CT derived 3-chamber Global Longitudinal Strain (10 cardiac phases). 67-year-old-male patient (see Fig. 2) with mild aortic stenosis with GLS of − 21% and an ejection fraction of 65%.
Supplementary file2 (MP4 4506 KB)

4D CT derived 4-chamber Global Longitudinal Strain (10 cardiac phases). 67-year-old-male patient (see Fig. 2) with mild aortic stenosis with GLS of − 21% and an ejection fraction of 65%.
Supplementary file3 (MP4 3959 KB)

4D CT derived 2-chamber Global Longitudinal Strain (10 cardiac phases). 74-year-old-female patient (see Fig. 3) with severe aortic stenosis with GLS of − 5% and an ejection fraction of 15%.
Supplementary file4 (MP4 3502 KB)

4D CT derived 3-chamber Global Longitudinal Strain (10 cardiac phases). 74-year-old-female patient (see Fig. 3) with severe aortic stenosis with GLS of − 5% and an ejection fraction of 15%.
Supplementary file5 (MP4 3832 KB)

4D CT derived 4-chamber Global Longitudinal Strain (10 cardiac phases). 74-year-old-female patient (see Fig. 3) with severe aortic stenosis with GLS of − 5% and an ejection fraction of 15%.
Supplementary file6 (MP4 3642 KB)

## Data Availability

No datasets were generated or analysed during the current study.
